# Anti-Sez6L2 antibody–associated autoimmune cerebellar ataxia: a rare case with implications of rituximab therapy

**DOI:** 10.1007/s10072-026-09017-0

**Published:** 2026-04-18

**Authors:** Ya Gao, Ruisheng Duan

**Affiliations:** 1 Department of Neurology I, Hebei Provincial People’s Hospital, No. 348, Heping West Road, Shijiazhuang, 050000 Hebei Province China; 2 Key Laboratory of Brain Network and Cognitive Impairment Disorders of Hebei Province, 1801 Building E, Zhongchu Plaza, 050000 Shijiazhuang, China

**Keywords:** Sez6L2, Autoimmune cerebellar ataxia, Rituximab, Rare antibody, Neuroimmunology

## Abstract

**Background:**

Anti-Sez6L2 (seizure-related 6 homolog-like 2) antibody–associated autoimmune cerebellar ataxia is an exceptionally rare neuroimmunological disorder, with only a limited number of cases reported to date. Its clinical spectrum and optimal therapeutic strategies remain incompletely characterized.

**Case presentation:**

We report a 77-year-old woman who developed a subacute-to-chronic progressive cerebellar ataxia accompanied by oculomotor abnormalities, a forward-leaning posture, and gait initiation failure.. Neuroimaging studies revealed no structural or neoplastic lesions sufficient to account for the neurological deterioration. Cell-based assays detected anti-Sez6L2 IgG antibodies in both serum and cerebrospinal fluid. The patient exhibited only limited and unsustained improvement following first-line immunotherapies, including corticosteroids and intravenous immunoglobulin. After confirmation of an antibody-mediated mechanism, treatment was escalated to B-cell–depleting therapy with rituximab, resulting in clinical improvement accompanied by a reduction in cerebrospinal fluid antibody titers.

**Conclusion:**

This case represents a rare presentation of anti-Sez6L2 antibody–associated autoimmune cerebellar ataxia and provides additional clinical and immunological evidence supporting the relevance of this antibody. Furthermore, it suggests that rituximab may represent a potentially valuable therapeutic option in patients with insufficient responses to first-line immunotherapies.

**Supplementary Information:**

The online version contains supplementary material available at 10.1007/s10072-026-09017-0.

## Introduction

Autoimmune cerebellar ataxias constitute a heterogeneous group of immune-mediated neurological disorders characterized by cerebellar dysfunction, a subset of which is potentially reversible with immunotherapy. Over the past decade, the identification of neuronal surface autoantibodies has substantially expanded the spectrum of immune-mediated ataxias and reshaped diagnostic and therapeutic paradigms, emphasizing disorders driven by antibody-mediated synaptic dysfunction [1]. However, the true prevalence of antibodies against targets like Sez6L2 remains unclear, as commercial and clinical testing is not widely available, potentially leading to underdiagnosis of this entity.

Seizure-related 6 homolog-like 2 (Sez6L2) is a transmembrane neuronal surface protein highly enriched in cerebellar neurons, particularly at synaptic structures. Experimental and translational studies have demonstrated that Sez6 family proteins play critical roles in synaptic organization, maintenance, and complement regulation, thereby providing strong biological plausibility for their involvement in immune-mediated cerebellar dysfunction [2–4]. Nevertheless, autoantibodies directed against Sez6L2 have been reported only exceptionally, and the associated clinical phenotypes, immunopathological relevance, and treatment responsiveness remain incompletely defined [5].

Given the extreme rarity of anti-Sez6L2 antibody–associated disease, standardized diagnostic criteria and therapeutic strategies have not yet been established. In this report, we describe a patient with anti-Sez6L2 antibody–associated autoimmune cerebellar ataxia, supported by systematic exclusion of alternative etiologies, comprehensive neuronal autoantibody profiling using cell-based assays, and detailed documentation of a stepwise immunotherapeutic course. Particular emphasis is placed on the clinical and immunological response to B-cell–depleting therapy with rituximab, consistent with current treatment paradigms for neuronal surface autoantibody–mediated neurological disorders [6].

## Case presentation

A 77-year-old woman with a history of type 2 diabetes mellitus and hypertension presented with a progressive gait disturbance over approximately seven months. Initial symptoms included slow walking speed, reduced step length, and postural instability, which gradually progressed to gait initiation difficulty, a forward-leaning posture, and recurrent falls. No prominent resting tremor was reported. There was no personal history of malignancy or autoimmune disease, and the family history was unremarkable (Table 1).

**Table 1:** Clinical and therapeutic timeline of the patient

Time Point	Event	Intervention	Clinical Status	Antibody Titer (Serum/CSF)
October 2024	Symptom onset	—	Progressive gait disturbance, postural instability	Not tested
November 2024	First hospitalization	High-dose corticosteroids, IVIG	Limited and transient improvement	CSF: 1:32 (at admission)
February 19, 2025	Second hospitalization (admission)	Diagnostic workup	Persistent gait initiation failure, postural instability, diplopia, truncal ataxia	Serum: 1:320/CSF: 1:32
February 25 – March 4, 2025	IVIG course, Rituximab initiation	Intravenous immunoglobulin, Rituximab (B-cell–depleting therapy)	Cough improved; clinical condition stable, B-cell depletion confirmed (CD19⁺: 0.42%), postural stability, enabling assisted independent standing and short-distance ambulation, Discharged	Serum 1:32/CSF 1:3.2
March – present	Follow-up period	—	No relapse; clinical condition stable	Regular monitoring of B-cell counts

Neurological examination revealed mild dysarthria with preserved consciousness. No psychiatric symptoms were reported by the patient or her family. Gaze-evoked diplopia and nystagmus were observed. Cerebellar signs were prominent, including impaired finger-to-nose and heel-to-shin testing, marked truncal ataxia. Muscle strength was mildly reduced, whereas muscle tone was largely normal. Deep tendon reflexes were preserved and symmetric, and no pyramidal signs or sensory deficits were detected.

### Investigations

#### Neuroimaging

**Fig. 1:**
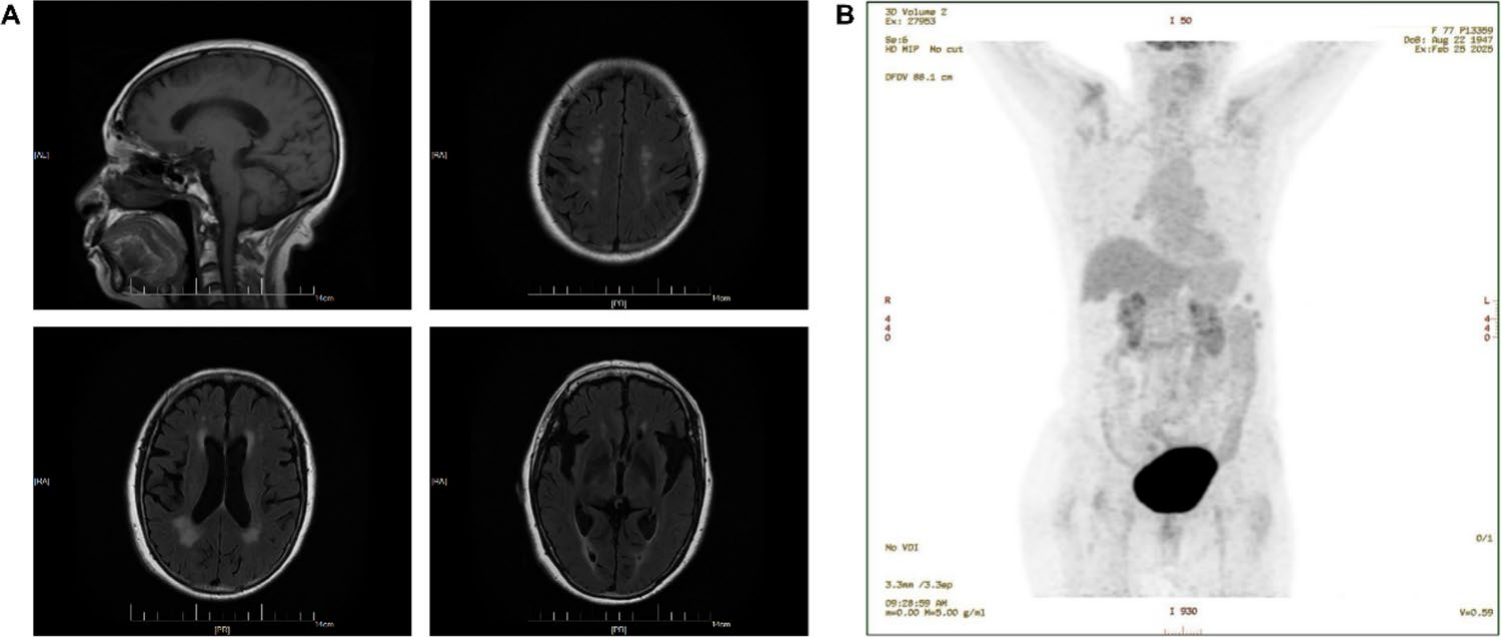
Neuroimaging and oncological screening findings. **A** Brain magnetic resonance imaging (MRI). Sagittal T1-weighted imaging shows preserved brainstem and cerebellar anatomy without mass lesions or cerebellar atrophy. Axial fluid-attenuated inversion recovery (FLAIR) images demonstrate scattered, symmetrical supratentorial periventricular white matter hyperintensities, consistent with nonspecific chronic microvascular changes and defining the upper extent of the lesions. Representative axial FLAIR images illustrate the typical distribution of these abnormalities. No acute ischemic, hemorrhagic, or vascular lesions involving the cerebellum or posterior fossa are identified. **B** Whole-body ^18F-fluorodeoxyglucose positron emission tomography (FDG-PET) shows no evidence of abnormal hypermetabolic lesions suggestive of an underlying malignancy. Overall, neuroimaging findings are insufficient to explain the patient’s progressive cerebellar syndrome

Brain magnetic resonance imaging (MRI) demonstrated chronic ischemic changes and mild cerebral atrophy without focal cerebellar lesions or acute vascular abnormalities sufficient to explain the progressive neurological symptoms (Fig. 1A).

Whole-body ^18F-fluorodeoxyglucose positron emission tomography/computed tomography (^18F-FDG PET/CT) revealed no abnormal hypermetabolic lesions suggestive of malignancy, arguing against a paraneoplastic etiology at the time of evaluation (Fig. 1B).

#### Cerebrospinal fluid analysis

Routine cerebrospinal fluid (CSF) examination was performed during both hospitalizations. Lumbar puncture revealed an opening pressure of 100 mmH₂O. CSF analysis showed mildly elevated protein levels (68.52 mg/dL during the first hospitalization and 67.34 mg/dL during the second hospitalization; reference range 15–45 mg/dL), with a weak positive Pandy's test. CSF cytology revealed a mild lymphocytic pleocytosis (6 cells/µL in the first and 4 cells/µL in the second hospitalization). Oligoclonal bands were not assessed. These findings, particularly the persistently elevated protein and mild pleocytosis, support the presence of intrathecal inflammation and are consistent with an immune-mediated process.

#### Autoantibody testing

**Fig. 2:**
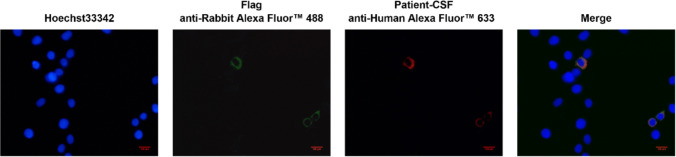
Detection of Sez6L2 antibodies in patient cerebrospinal fluid (CSF) using the cell-based assays. A plasmid expressing Sez6L2 with FLAG tag was constructed and transfected into HEK293T cells. Cells were incubated with FLAG antibody (green) and patient CSF (red) as primary antibodies. Cells expressing Sez6L2 protein were recognized by both FLAG antibody and patient CSF and showed co-localization, whereas cells not expressing Sez6L2 protein were not recognized by either antibody

A comprehensive autoimmune cerebellar syndrome antibody panel was systematically assessed using standardized cell-based assays (CBA), performed by Tianjin Enjis Medical Testing Laboratory (Tianjin, China), covering a total of 33 neuronal and paraneoplastic autoantibodies (Supplementary Table 1). Anti-Sez6L2 IgG antibodies were detected in both serum and cerebrospinal fluid (CSF) using cell-based assays. At baseline, prior to any immunotherapy, the serum titer was 1:320 and the CSF titer was 1:32. All other serum antibodies tested were negative. The specific binding of patient CSF antibodies to Sez6L2 was confirmed by a cell-based assay, as shown in Fig. 2. The complete list of antibodies included in the serum testing panel is provided in the Supplementary Material.

### Diagnosis and treatment

#### Diagnosis

Based on the subacute-to-chronic progressive cerebellar ataxia with oculomotor abnormalities, a forward-leaning posture, and gait initiation failure (features overlapping with parkinsonism), the absence of explanatory structural or neoplastic lesions on neuroimaging, and the detection of anti-Sez6L2 antibodies in both serum and CSF, the patient was diagnosed with anti-Sez6L2 antibody–associated autoimmune cerebellar ataxia.

#### Therapeutic strategy and stepwise response

During the first hospitalization, when specific antibody results were not yet available, the patient received first-line immunotherapies, including high-dose corticosteroids followed by tapering and intravenous immunoglobulin (IVIG), based on the suspected autoimmune etiology. These treatments resulted in only limited and transient improvement, with persistent gait initiation difficulty and postural instability.

During the second hospitalization, approximately 10 months after symptom onset, anti-Sez6L2 antibodies were confirmed. Repeat testing at this time point, prior to rituximab initiation, showed that antibody titers remained elevated (serum 1:320, CSF 1:32), and malignancy was reasonably excluded. Given the insufficient response to first-line immunotherapy and the presumed antibody-mediated pathogenesis, B-cell-depleting therapy with rituximab was initiated.

Following rituximab treatment, the patient experienced gradual improvement in gait initiation and postural stability, enabling assisted independent standing and short-distance ambulation. Follow-up immunological evaluation demonstrated marked depletion of peripheral CD19⁺ B cells. Analysis of anti-Sez6l2 antibodies showed that the anti-Sez6l2 antibody titer in CSF decreased from 1:32 to 1:3.2, and the titer in serum decreased from 1:320 to 1:32, paralleling the clinical improvement. No serious adverse events or severe infections were observed during follow-up.

## Discussion

Anti-Sez6L2 antibody–associated autoimmune cerebellar ataxia is an exceptionally rare neurological disorder, but accumulating evidence suggests that it represents a distinct subtype within the expanding spectrum of neuronal surface autoantibody–mediated diseases rather than an incidental serological finding [1, 5]. Reported cases consistently describe a predominantly progressive cerebellar syndrome, frequently accompanied by oculomotor abnormalities and, in some patients, parkinsonian or cognitive features, indicating a broader clinical phenotype than isolated ataxia alone [1, 5]. Neuroimaging findings are often nonspecific or unremarkable, supporting the concept that synaptic dysfunction rather than overt structural damage may underlie the clinical manifestations, particularly in early or active disease.

In the present case, extensive neuroimaging did not reveal structural or metabolic abnormalities sufficient to explain the progressive neurological deficits, thereby strengthening the exclusion of degenerative, vascular, and paraneoplastic etiologies. The identification of anti-Sez6L2 IgG antibodies in both serum and cerebrospinal fluid using a standardized cell-based assay, together with the systematic exclusion of other neuronal and paraneoplastic antibodies, provided robust immunological support for an antibody-mediated cerebellar syndrome. Importantly, the biological plausibility of anti-Sez6L2 autoimmunity is supported by foundational and subsequent experimental studies demonstrating that Sez6 family proteins are neuronal surface molecules enriched at cerebellar synapses and involved in synaptic organization, maintenance, and complement regulation, thereby providing a mechanistic link between antibody binding and cerebellar dysfunction [2–4].

The clinical phenotype in our patient extended beyond pure cerebellar ataxia to include features often encountered in atypical parkinsonian disorders, namely a forward-leaning posture and pronounced gait initiation failure. This overlap is clinically significant, as it broadens the differential diagnosis and poses a challenge in distinguishing autoimmune ataxias from neurodegenerative conditions, particularly in older adults. Given the patient's age (77 years) and the progressive, albeit subacute, course, disorders such as Progressive Supranuclear Palsy (PSP) and Multiple System Atrophy (MSA) were initially considered. The Richardson's syndrome variant of PSP, for example, can present with early postural instability and gait difficulties [7, 8]. However, the presence of prominent cerebellar signs (such as appendicular ataxia and nystagmus) from early in the disease course, coupled with the absence of a vertical supranuclear gaze palsy or early cognitive dysfunction, argued against a typical PSP presentation. Similarly, the lack of prominent dysautonomia (e.g., orthostatic hypotension, urinary incontinence) and the absence of characteristic MRI findings (like the 'hot cross bun' sign or putaminal atrophy) made a diagnosis of MSA-cerebellar type less probable [9, 10]. The subacute progression and the lack of an alternative explanation prompted a comprehensive search for autoimmune etiologies. The detection of anti-Sez6L2 antibodies not only provided a unifying diagnosis but also highlighted the importance of considering treatable autoimmune causes in patients presenting with rapidly progressive 'atypical' parkinsonism or cerebellar syndromes [11–13], as failure to do so may lead to a misdiagnosis of a primary neurodegenerative disease.

Beyond associative clinical evidence, recent experimental data have further strengthened the pathogenic concept by showing that Sez6L2-directed autoimmunity can induce cerebellar dysfunction and ataxia-like phenotypes in vivo, lending causal support to the role of anti-Sez6L2 antibodies rather than considering them mere epiphenomena [2, 4]. Collectively, these findings position anti-Sez6L2 antibodies within the group of functionally relevant neuronal surface autoantibodies implicated in immune-mediated cerebellar disorders [1].

From a therapeutic perspective, no standardized treatment strategy has been established for anti-Sez6L2 antibody–associated disease due to its rarity. Nevertheless, experience from neuronal surface autoantibody–mediated neurological disorders suggests that first-line immunotherapies such as corticosteroids and intravenous immunoglobulin often yield incomplete or transient responses, whereas escalation to B-cell–targeted therapy may be beneficial in selected patients [6]. Our observation aligns with a previous report by Mehdiyeva et al. [6], who also described clinical improvement with rituximab in a patient with anti-Sez6L2-associated ataxia, suggesting a consistent treatment response pattern. In line with these observations, previously reported cases of anti-Sez6L2–associated disease and related syndromes have described clinical improvement following more intensive or sequential immunotherapeutic approaches, supporting a stepwise, mechanism-based treatment paradigm [5, 6].

In addition, paraneoplastic associations have been documented in anti-Sez6L2 autoimmunity, including cases in which neurological improvement paralleled tumor-directed therapy, underscoring the importance of systematic malignancy screening and longitudinal surveillance when this antibody is identified [5]. Within this evolving framework, the present case contributes additional clinical and immunological evidence supporting anti-Sez6L2 antibody–associated autoimmune cerebellar ataxia and illustrates the pragmatic application of rituximab as a stepwise escalation strategy guided by diagnostic clarification and treatment response, consistent with current concepts in neuronal surface autoantibody–mediated neurological disorders [1, 6].

## Conclusion

Anti-Sez6L2 antibody–associated autoimmune cerebellar ataxia is an exceptionally rare, and likely under-recognized, neuroimmunological disorder, partly due to the limited availability of specific antibody testing. This case supports that, in patients presenting with progressive cerebellar ataxia and unremarkable neuroimaging, comprehensive neuronal autoantibody testing—including anti-Sez6L2—should be considered after exclusion of alternative etiologies. From a therapeutic standpoint, our observations suggest that when first-line immunotherapies yield insufficient or unsustained responses, a stepwise escalation strategy incorporating B-cell–depleting therapy such as rituximab may be a reasonable option. While treatment decisions must remain individualized, this approach may help guide clinical management and follow-up in future patients with suspected anti-Sez6L2 antibody–associated autoimmune cerebellar ataxia.

## Supplementary Information

Below is the link to the electronic supplementary material.

Supplementary file1 (DOCX 20 KB)

## Data Availability

The datasets used and/or analysed during the current study are available from the corresponding author on reasonable request.
