# Supplementary material for: Anti-Sez6L2 antibody–associated autoimmune cerebellar ataxia: a rare case with implications of rituximab therapy
Source: Neurol Sci. 2026 Apr 18;47(5):436. doi: 10.1007/s10072-026-09017-0 (PMC13090289; doi:10.1007/s10072-026-09017-0)
Supplement: Supplementary file 1 — Supplementary file1 (DOCX 20 KB) [file 10072_2026_9017_MOESM1_ESM.docx]

**Supplementary Table 1. Comprehensive Autoantibody Testing for Autoimmune Cerebellar Syndrome**

| Category | Autoantibody | Immunoglobulin | Method | Result |
| --- | --- | --- | --- | --- |
| Classical paraneoplastic antibodies | HuC | IgG | Cell-based assay | Negative |
|  | HuD | IgG | Cell-based assay | Negative |
|  | Yo (CDR2) | IgG | Cell-based assay | Negative |
|  | Yo (CDR2L) | IgG | Cell-based assay | Negative |
|  | Ri (Nova1) | IgG | Cell-based assay | Negative |
|  | Ri (Nova2) | IgG | Cell-based assay | Negative |
|  | CV2/CRMP5 | IgG | Cell-based assay | Negative |
|  | Amphiphysin | IgG | Cell-based assay | Negative |
|  | Ma2 | IgG | Cell-based assay | Negative |
|  | Ma1 | IgG | Cell-based assay | Negative |
|  | PCA-2 | IgG | Cell-based assay | Negative |
|  | ANNA-3 | IgG | Cell-based assay | Negative |
|  | SOX1 | IgG | Cell-based assay | Negative |
| Cerebellar/ ataxia-associated antibodies | **Sez6L2** | **IgG** | **Cell-based assay** | **Positive** |
|  | Tr (DNER) | IgG | Cell-based assay | Negative |
|  | Zic4 | IgG | Cell-based assay | Negative |
|  | PKC | IgG | Cell-based assay | Negative |
|  | TRIM46 | IgG | Cell-based assay | Negative |
|  | Homer-3 | IgG | Cell-based assay | Negative |
|  | ITPR1 | IgG | Cell-based assay | Negative |
|  | CARP VIII | IgG | Cell-based assay | Negative |
| Neuronal surface/ synaptic antibodies | GAD65 | IgG | Cell-based assay | Negative |
|  | ATP1A3 | IgG | Cell-based assay | Negative |
|  | ARHGAP26 | IgG | Cell-based assay | Negative |
|  | AP3B2 | IgG | Cell-based assay | Negative |
|  | Septin-5 | IgG | Cell-based assay | Negative |
|  | Neurochondrin (NCDN) | IgG | Cell-based assay | Negative |
|  | mGluR1 | IgG | Cell-based assay | Negative |
|  | GluR2 | IgG | Cell-based assay | Negative |
|  | mGluR2 | IgG | Cell-based assay | Negative |
|  | GluK2 | IgG | Cell-based assay | Negative |
|  | RGS8 | IgG | Cell-based assay | Negative |
| Other immune-mediated neurological antibodies | KLHL11 | IgG | Cell-based assay | Negative |
